# Self-employment, illness, and the social security system: a qualitative study of the experiences of solo self-employed workers in Ontario, Canada

**DOI:** 10.1186/s12889-023-15471-8

**Published:** 2023-04-04

**Authors:** Tauhid Hossain Khan, Ellen MacEachen, Stephanie Premji, Elena Neiterman

**Affiliations:** 1 School of Public Health Sciences, University of Waterloo, Waterloo, ON Canada; 2 Department of Sociology, Jagannath University, Dhaka, Bangladesh; 3 School of Labour Studies, Department of Health, Aging and Society, McMaster University, Hamilton, ON Canada

**Keywords:** Self-employment, Health, Illness, Injury, Social security, Social support, Social protection, Covid-19, CERB

## Abstract

**Background:**

Today’s labor market has changed over time, shifting from mostly full-time, secured, and standard employment relationships to mostly entrepreneurial and precarious working arrangements. Thus, self-employment (SE) has been growing rapidly in recent decades due to globalization, automation, technological advances, and the recent rise of the ‘gig’ economy, among other factors. Accordingly, more than 60% of workers worldwide are non-standard and precarious. This precarity profoundly impacts workers’ health and well-being, undermining the comprehensiveness of social security systems. This study aims to examine the experiences of self-employed (SE’d) workers on how they are protected with available social security systems following illness, injury, and income reduction or loss.

**Methods:**

Drawing on in-depth interviews with 24 solo SE’d people in Ontario (January – July 2021), thematic analysis was conducted based on participants’ narratives of experiences with available security systems following illness or injury. The dataset was analyzed using NVIVO qualitative software to elicit narratives and themes.

**Findings:**

Three major themes emerged through the narrative analysis: (i) policy-practice (mis)matching, (ii) compromise for a decent life, and (iii) equity in work and benefits.

**Conclusions:**

Meagre government-provided formal supports may adversely impact the health and wellbeing of self-employed workers. This study points to ways that statutory social protection programs should be decoupled from benefits provided by employers. Instead, government can introduce a comprehensive program that may compensate or protect low-income individuals irrespective of employment status.

## Background

Today’s labor market continues to evolve, and self-employment (SE) has become a prevalent non-standard, precarious, and contingent work arrangement internationally [1–3]. By SE, we refer to individuals who work for themselves instead of working for others (employees). Some of these may work alone, while others may have small business with or without employees. SE is a diverse work arrangement, encompassing occupations ranging from highly paid professionals to low-skilled workers. SE appears in different forms and contours in the current digital age than it did 50 years ago following a paradigm shift from managerial capitalism (employer-employee relations) to entrepreneurial capitalism (own boss) [4, 5]. Precarious work, including SE, has been growing rapidly in recent decades due to globalization, automation, technological advances, socio-demographic changes, neoliberal policies, and the decline of manufacturing industries [1, 3, 6, 7]. The International Labour Organization (ILO) estimated that non-standard employment accounts for more than 60% of workers worldwide [8]. In Canada, 2.9 million people were self-employed (SE’d) in 2018, which is more than double the number in 1976 [9]. Overall, SE’d workers make up 15% of the workforce in Canada [9], 10% of the Australian workforce [6, 10, 11], and 15% of the workforce in Europe [12]. The rise of the ‘gig economy’, together with the breakdown of traditional employment systems that provided secure, lifetime positions with stable income, contributes to this SE trend [3, 13–15]. In tandem, globally, SE’d workers are excluded from most social security supports, such as income support when ill or injured, which are provided to employees via systems of employers’ and workers’ employment contributions [16–18]. The ILO’s (2020) study of G20 countries found many social protection coverage gaps for SE’d workers [19]. Against this backdrop, it is unclear how and if existing workers’ support and protection systems have adapted to new labour market situations and expectations [3].

SE’d workers have often been depicted in research literature as a distinct group of homogeneous people who enjoy good health, the freedom of being their own boss, flexible working hours and who do not rely on the government (e.g., social security protection). They are described as having a higher level of job satisfaction, quality of life, and opportunities for work-life balance than employees [12, 20–22]. They have a reputation for taking on significant personal risk in order to build their company and create jobs for others [12, 13, 23, 24]. However, these depictions do not reflect the recent reality of the SE’d, in which a large number of SE’d workers in a given society are forced to do so due to unemployment, a lack of alternatives, and financial challenges [12, 23, 25–30]. These studies highlight a strong relationship between precarious jobs and poor health outcomes [31, 32] and numerous social costs [23, 33]. When compared to salaried workers, SE’d workers are at a higher risk for diseases (physical and mental) [23, 33, 34]. SE’d people in some types of work face significant job demands and workloads (e.g., farmers), self-exploitation (drudgery), and isolation due to working alone, lack of social protection (e.g., health insurance), and anxiety about financial matters due to volatile income [23]. In addition, the dominant narrative that the SE’d tend to be healthier than salaried employees [23, 34–36] overlooks the ‘selection effect’ [23]. That is, these studies might be biased by the ‘healthy worker effect’ in which only healthy workers are studied or healthier individuals self-select into SE [23]. In contrast to depictions of SE’d as homogenous, the diversity of SE’d workers was described by the Law Commission of Ontario (2012), which noted that: “the experiences and vulnerabilities of this group range from billionaire entrepreneurs to taxi drivers working 90 hours a week simply to pay their bills and includes many people who are gaining income from SE activity alongside their main job” (LCO, 2012: 75). Therefore, SE does not always mean self-sufficiency.

In Canada, the federal, provincial, and territory governments regulate labour and employment legislations, with the provinces and territories regulating the majority of employment-related matters. Some Canadian social security programs, such as Employment Insurance, are administered federally but many programs, including workers’ compensation benefits and disability income support programs, are administered at the provincial level, and these programs differ province-by-province [18, 37–40]. In Ontario (Canada), with the exception of temporary COVID-19 pandemic measures, SE’d workers are supported under basic welfare support programs (Ontario Works), income support for people with disabilities (Ontario Disability Support Program), health insurance (Ontario Health Insurance Plan, or OHIP), workers’ compensation (in Ontario, called the Workplace Safety and Insurance Board) which was required for SE’d construction workers, retirement pension plan (Canada Pension Plan), and income support to cover child and caregiving employment absences (Employment Insurance Special Benefits) [17]. However, these schemes are not fully accessible to SE’d workers due to challenges with accessibility, unaffordable premium rates, and administrative complications.

Although a growing body of research examines SE’d workers’ health and well-being, social mobility, and racial and gender discrimination [2, 41–45], as well as their status as precarious workers, entrepreneurs, and small business owners, very few studies examine social security and support systems to which SE’d workers have access [1, 7, 28]. Although formal or statuary support systems concerning SE’d workers have received scholarly attention, their overarching foci have been based on policy-level analysis, occupational health and safety of precarious workers, or on entrepreneurs and small business owners and based on census data [46–48].

Very little research has shed light on the formal social support systems using a holistic perspective; that is, how SE’d experience and navigate these support systems following their illness, injury, and income reduction/loss. Moreover, few studies have used qualitative methods to investigate the experiences of SE’d workers, and specifically, we have very thin knowledge about solo - self-employed workers in this context. Importantly, although SE is not new, work within this sector has expanded and changed in tandem with the changing labour market. In the EU, more than two-thirds of all SE’d workers are solo SE’d (without employees). Mounting socioeconomic and commercial drives are responsible for this rise, including new business models, organizational decentralization, and institutional deregulation (e.g., IT experts, business consultants, freelancers)[49]. Similar to other forms of SE’d, solo SE’d workers are increasingly associated with precarity in terms of income and social security [40]. Given this backdrop, the limited scholarly attention to the precariousness of solo-self-employed workers in terms of their access to statutory support systems, following their illness, injury or income loss/reduction, this paper aims to fill these gaps by examining solo SE’d workers’ experiences of navigating formal supports systems, reflecting on how they are protected with available social security systems following illness, injury, income reduction and/or loss.

## Methodology

### Study design

Given our interest in SE’d workers’ narratives, including their personal experiences, perceptions, and practices of navigating formal support systems following their illness, injury, and income reduction or loss, this study adopted a qualitative methodological approach. Consistent with this approach, we reflected on the narratives our participants provided using an interpretative paradigm, which focuses on the understanding of phenomena through meanings people bring to them [50–53]. Accordingly, our study followed an interpretive narrative approach, which examines stories/narratives for how we interpret our everyday experiences [40, 54]. This approach helped to unpack the underlying meanings embedded in SE’d workers’ stories, including everyday practices and experiences situated in a larger cultural context. The study was approved by the Research Ethics Board of the University of Waterloo, Canada.

### Participants, sampling, and recruitment

To be included, participants in this study had to meet the following criteria: solo SE’d workers (i.e., no employees), aged 18 years and older, experience of illness or injury (work-related or not), main income is from self-employment, and (due to researchers’ language limitations) fluent in English (Table 1). The study included similar numbers of men and women and their ages ranged from 21 to 62 years. Income levels varied greatly, with one participant earning $200k/year and the lowest-earning participant earning only $25k/year. Workers had a range of education, including college diplomas (vocational training) and university degrees. Participants were recruited from Ontario, Canada using different social media platforms: LinkedIn, Facebook, Kijiji, Twitter, and Tumblr[55]. From among eligible participants, we selected participants purposively for information-rich and heterogeneous cases [52, 53, 56]. The lead author (TK) interviewed 24 solo SE’d workers using audio/video conferencing with Zoom and WhatsApp between January and July 2021. Interviews lasted an average of 1.10 h.

**Table 1:** Participant characteristics

Pseudonym	Gender	Age	Education	Type of SE’d work	Type of illness/injury	F. Income (CAD)/Year
1.Habibur	M	22	College diploma	Uber driver	Depression Leg fracture	50 K
2.Tasmina	F	32	College diploma	Home childcare	Flu/ fever	50 K
3.Emma	F	36	Undergraduate degree	Catering	Pneumonia	25-50 K
4.Mamun	M	45	Graduate degree	IT consultant	Spinal injury	45 K
5.Zayan	M	22	College diploma	Food delivery: Door dash Skip dish	Breaking ankle	100 K
6.Ruby	F	42–47	Graduate degree	Rotary Public commissioner	Depression Stress, Obesity	25-50 K
7.Patrick	M	62	Undergraduate degree	Actor, catering	Knee injury	50-100 K
8.Sarah	F	54	Graduate degree	Property manager	Stomach pain	50-100 K
9.Sumon	M	22	College diploma	Food delivery	Breaking right hand	25-50 K
10.Mary	F	46	High school	Fashion design	Sjogren syndrome	< 25 K
11.Faria	F	21	Undergraduate degree	Beautician	ADHD	25-50 K
12.Remi	F	45	College diploma	Financial advisor	Asthma, Covid-19	50 K-10 K
13.Sarika	F	50	High school	Cleaner	Sleep disorder	25-50 K
14.Scott	M	50	College diploma	Construction	Arthritis	50-100 K
15.Ander	M	25	Postgraduate diploma	Online business/ E-commerce	Anxiety, stress, depression	25-50 K
16. Bob	M	33	College diploma	Singer, DJ	Anxiety, stress Back pain	25-50 K
17.Jane	F	33	Undergraduate degree	Actor, Writer	Nervous system disorder	130 K
18.Jimmy	M	35	Graduate degree	Data analyst	Regular migraine	200 K
19. Paul	M	32	College diploma	Electrician	Backbone Injury	50 K
20. Ayla	F	35	College diploma	Grocery business	Cardiology ADHD	50-100 K
21.Miller	M	24	Undergraduate degree	Music trainer, musician	Leg injury	50 K
22.Mila	F	35	Graduate degree	Tailoring	Backpain, Fatigue	50-100 K
23.Arnob	M	30	Graduate degree	Debate /public speaking trainer	Anxiety, stress, burn injury, depression,	25-50 K
24.Pablo	F	26	College diploma	Financial advisor	Stress	25-50 K

### Data collection

As this study involves soliciting solo SE’d workers’ personal experiences including culturally sensitive information (e.g., income, sickness, personal family lives), an in-depth interview approach was selected to give time and space to each person to explain their situation. In our research, we focused on disability support program, health insurance plan, workplace safety and insurance, pension plan, and employment insurance benefits, and defined formal support systems for the SE’d worker as those that include services provided by the government.

A semi-structured interview guide was used, which was informed by literature and discussion with the research team/committee. We used a combination of questions and probes (follow-up questions) to achieve breadths of coverage across the following key topics: (a) stories about their work-related experiences; (b) stories about their illness, injury or income reduction/loss; (c) their use and knowledge of social security programs available to them in relation to the experience of health and illness. To ensure an informed discussion, the interviewer informed all participants orally of Ontario social security programs available to SE’d and asked for their views of these programs. Interviews were audio-recorded and transcribed verbatim by two professional transcriptionists. Along with reflexive journal/diaries, detailed field notes were taken after each interview to describe encounters, including the immediate impressions and context, and analytic insights.

### Data analysis: thematic narrative analytical approach

Following Reissman’s (2008) Narrative Thematic Analytical Approach (NTAA), we sought to understand in detail the experiences and practices of SE’d workers as told stories (narratives) pertinent to accessing social security systems following illness and injury [41, 42]. In this context, unlike another type of narrative analysis, TNAA is suitable because it focuses on “what content a narrative communicates [what is told or spoken], rather than precisely how a narrative is structured to make points” [57, p.81]. The analysis involved several phases: reading the transcripts several times, developing a codebook, developing themes and subthemes, and identifying core narrative elements associated with each theme. Of importance, data analysis followed a mixing of deductive and inductive coding. A codebook of 10 codes was created in this context. These codes were predetermined codes from the previous literature, and research objectives/questions reflected issues recognized or assumed during interviews by the lead researcher. Using NVivo, the data sets were re-arranged in terms of the codebook. These codes helped us reflect on the overall patterns of the data, including identifying common themes that yield many descriptive themes. We then (re)viewed these descriptive themes again and developed more analytical themes by grouping them together, moving back and forth between descriptive and analytical themes, using a word document. This facilitated a higher level of abstraction and theorizing the interpretation of the research findings and the function they serve. Thus, we found some analytical and abstract themes through several reviews and re-reviews of the long list of (descriptive) themes through which the major /key themes emerged. Thus, my analysis led to the development of three key major themes and several sub-themes, as discussed below.

### Findings

This paper discusses participant stories about their interactions with government support and social security systems following their illness, injury, and income reduction and/or loss. This section begins by discussing a theme, where participants discussed their knowledge of available government support systems and reflected on their experiences dealing with and navigating these systems in terms of their work, health, illness/injury, and income loss/reduction. Then this section moves on to examine participants’ experiences, and related views of SE’d workers concerning opportunities provided by the existing social security systems, including their shortcomings and strengths.

### Policy-practice (mis)matching

SE’d workers in our study described their understandings of the benefits and drawbacks of different government-regulated social protection systems and policies. They reflected on their experiences of navigating and dealing with these available systems, including medical benefits, income supports, and other government supports (Table 2).

**Table 2:** Social Security Supports to SE’d workers in Ontario, Canada

**Supports that cover /required for all SE’d workers**	
• Ontario Disability Support Program • Ontario Works • Ontario Health Insurance Plan (OHIP) • Workplace Safety and Insurance Board (workers’ compensation, among SE’d, required participation for construction workers only) • Canada Pension Plan (Federal) • Canadian Emergency Response Benefit (CERB) (Federal) • OHIP+ (for age 24 and younger)	
**Supports that are available to SE’d workers only if they opt in and pay a premium**	
• Employment Insurance Special Benefits (Federal) • Workplace Safety and Insurance Board (workers’ compensation, for all occupations except construction)	

The first step for SE’d workers gaining access to government supports was awareness of the system, including their different requirements and procedures [58]. This social security system literacy was critical for SE’d workers as they made important decisions about opting into a scheme based on the benefits and drawbacks of each scheme. In our study, several participants did not know about government support systems available for them in Canada/Ontario (Table 2), and some knew about these schemes only partially. For example, Jimmy, a data analyst, was unaware of available government support systems for SE’d: “But beyond that [savings] I say no […] government support that I am not aware of any at least any one thing that is specific to SE’d”. Similarly, Sarika, a cleaner, was surprised when asked about government support systems available for SE’d workers:

Oh! government support? […] for solo self-employed. I’m not aware of any I know if I had employees that then I could more easily get, like, group benefits at a lower cost.

As they were SE’d, many participants in this study believed that the government could do nothing for them; they were responsible for their own protection. It is possible that lack of knowledge about these programs among some participants could have been attributed to the fact that they were confident about their savings; and, indeed, some participants believed that they could support themselves with their savings when ill or facing a reduction of income.

The majority of participants were aware of options for purchasing private insurance (out of pocket). Some of them, whose annual income above 50k, described purchasing private insurance for critical and chronic diseases, retirement benefits, and life insurance, regardless of income ceiling, in this study, some participants described purchasing private insurance for critical illness.

In addition to lacking knowledge about government support systems, some participants also misunderstood what supports were available through the government systems. For example, Zayan, a young man studying at the undergraduate level, said he had heard about ‘unemployment insurance’ for SE’d workers, despite no unemployment insurance (regulated by federal and Ontario Government) being available in practice for them. These types of system misinformation reveal how SE’d workers had sometimes not looked into the availability of support systems.

Most Canadians feel proud of their global reputation for universal health coverage [44]. The Ontario Health Insurance Plan (OHIP) is very well known and available to all citizens irrespective of working status, and SE’d workers are no exception. However, participants in our study described concerns about the scope of health insurance coverage. It is not fully comprehensive and does not cover many therapies. It fully excludes prescriptions, chiropractic treatment, massage, eye exams and dental treatment (OHIP + program covers prescribed medicines for people who are under 24 years and over 65 years old). Along with the impacts on SE’d workers, it is noteworthy that this limited nature of health insurance also affects regular employees who are in jobs (usually low waged) with no health benefits. This study found that many of the SE’d interviewed were not satisfied with limited coverage provided by the health insurance. Sumon, a delivery worker, noted on the issue of partial coverage of this health insurance:

No. It was not enough […] the insurance [EI] and the provincial health card doesn’t cover the most of it. Still, you have to pay from your pocket. I had to put the plaster/bandages that put in my hand when I broke my bone. So, I have to change it for 4 to 5 times, and I have to pay each and every time. Sorry I changed it 5 times, but insurance covered the price for two time. So, all together it is 600$. However, my insurance paid 200$ only.

Participants in our study stressed that they had to spend their own money on health insurance exclusions, which placed a burden on their finances. Ironically, in general, many low-wage SE’d workers rely heavily on health insurance for their health and wellness. For non-health insurance-covered health needs (e.g., prescriptions), they do not have employer-provided insurance and often cannot afford private insurance.

Many participants in our study were familiar with government support systems, such as Employment Insurance Special Benefit (EISB), which was introduced to SE’d workers in 2010 [45] and provides them with income support related to leave for parental care, sickness, compassionate care, and family caregiving after registering and paying at least one year of monthly premiums, the premium is as much as the rate of regular employment insurance (1.58% of annual income) and it is changeable year to year assessed by Canada Revenue Agency (CRA) [40]. Many SE’d in our study did not trust these government-regulated schemes. With respect to income support benefits for sickness and family care (EISB), they described not being able to rely on this system because they had previous dissatisfying experiences with Ontario government-provided benefits programs in terms of procedure of claiming benefits, paperwork, premium systems, and other administrative issues. Remi, a 45-year-old financial advisor, reflected on her experience with claims to Employment Insurance (EI) before entering into SE:

[in response to EISB as government regulated programs] I would probably not. I’m paid into EI [employment insurance] many years, jobs before I’m paid into. One time I had to claim. I don’t trust the government …. they asked [for] lots of documentations, which I was not in that state to provide them, was too complicated and convoluted, mentally and physically I was not ok to meet their requirements.

In our study, no participants opted into income support benefits for sickness and family care (EISB). Most of the SE’d workers stated that they could not afford the premiums. In addition, some used the metaphor of a “loan program” when describing their experience of this program; that is, they questioned the point of getting this insurance if the premium and their amount of monthly income or savings are equal. For example, Sarika, a SE’d cleaner, was wary of the premiums: “But again, it would depend on what the premiums are if it’s [financially] worthwhile”. As well, Scott, a construction worker, and Jane, an actor and writer, expressed similar concerns about premiums. Scott saw the income support benefits for sickness and family care as a loan instead of a benefit program: “It’s more of a loan program as far as I’m concerned … it gives me $900/month, but I pay taxes $900/month for [premiums]”. Jane similarly didn’t see the benefit:

I just found that it wasn’t worth it like your premiums for the same number of benefits that you got. so, I don’t know why you would have that insurance when you were essentially just paid monthly for the exact same thing that you are getting it back.

Our discussion with SE’d workers raised the question of whether programs, such as income support benefits for sickness and family care, should be mandatory or optional. Some participants favored compulsory, while others preferred optional. In fact, the perceived necessity of opting into social security programs (that require premiums out of pocket) is likely to be influenced by the income level, type of SE’d work, and opportunity for informal or family supports. For example, although Jane, a 33-year-old actor and writer, has a family income of more than 100k, she strongly believes that it should be mandatory for their protections and safety because the arts industry, where she works, has volatility in terms of income and amount working hours:

I think it should be mandatory to be honest because, yes, when you’re SE’d a lot of people [who] have trouble, paying into something like that. But if something were to happen […] they really need that protection and I think a lot of people don’t think that. They’re just thinking about, you know, the invoice, the money that’s coming in and they’re thinking about today. They’re not thinking about down the line, you know they are [potentially] heading to an uncertainness. Nobody knows what may happen.

Similarly, Patrick also showed interest towards EISB or something like this: “Yes. Because like I said before […] I almost went bankrupt twice. So, I’m always unprotect, the money I would that I put aside for savings, not guaranteed. I would have whatever I would have looked into it and paid into special EI. I never know it existed, never. I never knew that existed Never heard of it”.

Unlike Scott and Jane, Sarah rather assuaged the concerns, but still confused, that he should opt into this as long as his monthly income is around 2000$: “depends on the cost my income is 2000$ on a month …I think it would depend on how much, how much right I have to pay, you know the determine if it is worth it or not”.

On the other hand, Sarika disagreed that programs should be made mandatory, as she felt that people have different perspectives, contexts, and needs. For instance, although her own annual earnings were low ($25k), she was in a dual-income household with support through her partner. She noted that others, like her, might not be in need of insurance if they have a dual source of income:

I don’t feel it should be mandatory because everybody’s circumstances are different. Like, you know … if I was married … I would have somebody else as financial to help as well. So, I don’t think it should be mandatory. But I think it should be … more known [campaign] so that people look into that more often.

Thus, Jane and Sarika’s conflicting views regarding adopting social security programs may have been, in part, derived from their different financial positionality as SE’d workers.

Ontario Works provides means-tested programs and is only available for people who have assets no more significant than the limits set out by the program. In the case of Ontario Disability Support Program (ODSP), a sub-program of Ontario’s basic welfare program, in addition to the financial ceiling requirement, people have to meet their administrative definition of disability. However, many of our participants found inclusion criteria for benefits were unrealistic and overwhelming. Remi, a financial advisor, described her non-use of ODSP services in this way: “I have disability insurance, myself disability insurance, however, there lots of conditions that needed be met in order to collect disability benefits.” She was disappointed that, to be eligible, she needed to be “absolutely disabled.” This disability benefit requirement of absolute disability was controversial for many SE’d workers in this study. Similarly, Mary, a fashion designer suffering from a long-term chronic disease, was aware of disability benefits and that they were not available for her:

“I know that ODSP [disability benefits] is available right now. It’s not available for me [due to eligibility criteria]. There are different community agencies. Like, if I was struggling with food and security more than I am currently, I can go to a food bank”.

SE’d workers who opt into workers’ compensation coverage and pay monthly premiums are eligible for income support when ill or injured at a rate of 85% of the worker’s net wages. However, for a SE’d person, this amount can be substantially smaller than their regular monthly income, which includes income to cover business as well as personal costs. Paul, a licensed electrician who subscribed to workers’ compensation insurance, discussed his benefit experiences. He had been working for less than six months when he fell in an accident at his workplace and injured his leg. Although he was receiving workers’ compensation income benefits, he was dissatisfied with the benefit amount because it was substantially less than his usual monthly income. In addition, he questioned the workers’ compensation calculation method, which he saw as unfair because it did not cover his overhead expenses:

[…] Its 20% of my gross income. And they have based that upon the average type, the average ah, invoicing that I did per month. Well. Ideally, I like every dollar that I could have made reimbursed. But you know, they have to take into consideration that, […] My ability to generate income is forecasted over the last year of my proof of income based on income tax, you understand. So, they cannot forecast that next month, I will make a million dollars when I can’t show that in the past. I made a million, right? is it acceptable based on probability of my ability to generate income. So, I have to be satisfied with their compensation [ though unfair].

With this reduced income, he had to adjust his spending in terms of groceries, transportation, and recreation.

As discussed above, a group of participants in our study did not take up government -provided formal support systems due, in part, to high premiums. Another group of SE’d workers could not afford premiums for private health and income insurance coverage and instead relied substantially on informal support systems. Several important issues emerged in this context. First, SE’d workers who could afford premiums preferred private insurance instead of government regulated schemes. Second, the SE’d workers, who could not afford private insurance, thought that government-group insurance, rather than private insurance, would be preferable because group insurance is relatively less expensive, as echoed in Sarika’s narratives:

Yeah, private [insurance was an option].… but it was even more expensive, like, it was crazy! So, I decided I would rather try to save the money myself first [because my income is inconsistent].

Jane suggested that some sort of government group insurance might help SE’d workers, as its premiums could be affordable. Sarika also reflected on how, for her, even private insurance was out of its reach because her hours were variable, and so she did not meet the eligibility criteria of three months of consistent income. As such, SE’d workers in this study indicated that to be helpful, insurance needed to be flexible because their income was unstable. In a nutshell, most participants in our study had information/knowledge gaps and misinformation regarding existing social security programs from which SE’d workers could opt into or opt out. In terms of health coverage, many of them had to spend out of pocket because the Ontario health insurance system did not cover some expenses, including dental and mental health services, or drugs. With respect to income support benefits for sickness and family care, even when participants were aware of the program, they could not afford it due to the high premiums. In addition, some participants described bureaucratic complications and limited trust in the government provided schemes. Overall, they saw social insurance as preferable to private insurance due to perceived lower premium rates.

Most participants in our study were highly engaged in discussing how they were supported by the Canadian Government-regulated emergency response programs during Covid-19. Their focus of discussion mainly centered on Canada Emergency Response Benefit (CERB) and a little on Canada Emergency Student Benefit (CESB). The COVID emergency support benefit provided lump sum income support ($500/week) based on some eligibility criteria, including people who had employment and/or self-employment income of at least $5,000 in 2019 or in the 12 months prior to the date of their application. The COVID student benefit provided $1,250/month to post-secondary students, and recent post-secondary and high school graduates who did not apply, receive, nor qualify, for the COVID emergency support benefit or employment insurance benefits for the same eligibility period. Most participants saw these programs as excellent (in their words: “fantastic,” “wonderful”) and as acknowledging SE’d workers as contributors to the economy. Mary, a fashion designer, noted how well Canadian COVID related support systems performed in comparison with those in the US:

I mean, Canada as a whole has done a great job in supporting its citizens through the pandemic. We’ve done our best […] Ah, you look at the United States, and they’ve given out how much? Very little […] and people are dying. They’re the [high] numbers in the States because people cannot go to work. But here, people who had their jobs canceled are still able to meet their basic needs? […] There’s so much more we can do to support people when they go through hard times, whether it’s a lifelong chronic illness or something acute that is distributable. We could do more for people.”

However, several participants critiqued the program for having vague eligibility criteria. They felt that the Canadian government’s request to COVID emergency support benefit recipients who did not meet eligibility criteria to repay benefits was an example of government mismanagement. This group of SE’d workers also argued that the government did not provide a sustainable solution to protect the incomes of SE’d workers. In addition, several participants raised a question about misuse of the system in the event that people are doing cash jobs and receiving benefits simultaneously. They witnessed friends and relatives who were not going back to jobs intentionally as they were getting $2000/month with the COVID emergency support benefit. Thus, some study participants felt that it was better to have no government-provided funds at all rather than to have a program open to misuse:

“Because […] system would be abused completely. You can even see it now that people [I know] … have traveled outside of Canada [while collecting these benefits]. They’re cracking down on them …. [but government should] not going to give them COVID relief [leading to] people go on vacation and are getting money”.

In a nutshell, participants saw the COVID emergency support benefit as helpful for the ‘really needy’ SE’ workers who abruptly lost their jobs and income. Even though it was launched during an emergency, they also argued that the program should have provided more clarity. Finally, our participants went on to discuss a sustainable protection system for SE’d during any time of financial distress, including pandemics, sickness, and natural disasters. Many participants focused on Universal Basic Income for workers, including SE’d people, arguing that this type of support is necessary for SE’d if they lose income or become sick or injured. In this context, although Jimmy, a 35-year-old data analyst, had a family annual income of 200k, he strongly supported the Universal Basic Income because he felt there was no guarantee that his health would always favor his ability to earn an income.

### Compromise for a decent life: a potential threat to health and wellness

As discussed above, most participants felt the available government-provided support systems did not sufficiently protect their incomes. When ill or injured, they had to rely substantially on limited savings or on programs such as the Ontario health insurance plan because, as SE’d, they had no employer-provided income support benefits or health insurance. In addition, many solo SE’d workers could not afford private insurance for health and income support. As a result, they were forced to compromise their living standards and fell into conundrums such as whether they should buy groceries or medicine. As such, many participants in our study stressed that they were often compelled not to take medication when ill in order to stay financially afloat. Even Scott, a SE’d construction worker and one of the top earners in our sample (up to $100k/year), reported not being able to afford medication:

“I don’t take medication either for it. So, because we can’t afford it […] Well, health care is free in Ontario, but medications, I can’t afford them. So, I get what I can do, when I can do”.

He further expressed his dismay with this lack of coverage: “It sucks because I have to live with the pain”. This participant had no savings and could not afford his required medications, which were not covered by the Ontario health insurance program.

Similarly, Ander, who ran an e-commerce business, noted the tension between food and medicine, “I would rather spend this much money on groceries rather than on medicine. However, medicine is important”. These compromises between health and a decent life are echoed in the narratives of Mary, a SE’d fashion designer, who has been wrestling to manage her chronic health problem by sometimes using undermined quality of health services:

I have to pay for my medication from my own pocket. I have to pay for my IV therapy, a small fee, because part of it is covered under OHIP [Ontario health insurance]. [have to pay for transportation, rheumatologist, neurologist] blood work is covered […] except what I need a special test every now and then tand it’s $60 […] I can’t afford them right now. Like an hour’s massages expensive $80 to $120. I don’t have money for that. So, I bought a massage pad to try to help ease those symptoms that massage would help. [seeking another way] they are colleges students […] massage therapy osteopath those kinds of things where they need people to practice on and they’ll do it for free.

Similarly, it was then challenging for Ruby, a rotary public commissioner, to buy medicine out of pocket, which was previously covered by government provided insurance. She echoed on this issue: “they are cutting healthcare more like, they used to cover the vitamin D test, I now pay for it every year as my doctor say; so, it could affect my health as long as I don’t”.

Several SE’d workers in our study asserted that their savings were not always sufficient to support their health and daily necessities following their illness or injury. In turn, they were compelled to depend on credit card loans. In this context, they were concerned about falling into a vicious cycle of loans and poverty. Mamun, a SE’d IT expert ($45,000.00/year), reflected on the issue of debt: “My savings was very poor …. […] not enough to support my unworked period. So, I had to charge my credit card a lot, and after [finding more] work, I have to pay those [bills]”. In a nutshell, though Scott (a construction worker) and Mamun (IT expert) were in a good position in terms of income, their financial concerns signaled economic uncertainty for SE’d workers.

### Equity in work and benefits: a call for social justice

In terms of equity or fairness for SE’d workers in relation to government support systems, many participants argued that they should be treated equally to the salaried workers in terms of social security system protections, as they are also contributing to the economy. Scott strongly raised his voice against this injustice by comparing SE’d with salaried and unionized workers:

“You know what need treat everybody equal. Just because I’m SE’d doesn’t mean that I’m not deemed as human as a person … We don’t have any protection as a self-employed person.”.

Jane, an actor, and writer, also raised a similar point about SE rights:

“Everyone has the right to have housing and food and you do not worry about those things. You wouldn’t have to worry about being hungry because worrying about those things or struggling with those things definitely contributes to not being able to work as hard when you’re self-employed”.

Faria, a beautician, called for paid sick leave for SE’d workers, suggesting that this would be justice for them:

“I think that is unfair, because If you are a worker or employed person in a company and a self-employed person, they are both work. So, I think having paid sick leave is fair for self-employed people [such as] ourselves”.

As such, most of the SE’d workers in our study called for justice in terms of social equality in accessing work and support systems provided by the government. By social justice we underlined on equality in relationships and restoring relationships, which exist “when relationships are such that each party has their rights to dignity, equal concern and respect satisfied” [59]. In this context, the state or government can play a pivotal and critical role as an agent of social justice, which is a commitment from the government to be open to and facilitate change in the current system to make the system workable [59]. Many SE’d workers in our study lived pay cheque-to-pay cheque, experiencing insecurity and precariousness in their lives.

## Discussion

While workers with regular employment relationships are protected with statuary and employer support systems, SE’d workers often slip through the cracks. In this context, our study reaffirms existing research findings that SE’d workers are left out of social security systems [3, 6, 14, 18, 19, 39, 48, 60]. This study revealed structural (premium affordability, lack of information, lack of SE ’d-focused support programs) and non-structural factors (e.g., lack of trust in government systems) that led to poor access to formal support for SE’d workers. Yet little has been documented in the existing literature regarding the formal support system’s effectiveness and accessibility, as experienced by SE’d workers. Findings presented in the paper thus contribute to this literature to fill these gaps.

In our study, SE’d workers described a conspicuous knowledge gap with respect to existing formal support systems. Previous studies have underlined why social security literacy is primary requirement for populations to avail the social security systems [58]. Why were SE’d workers in our study not aware of the formal support systems? Two groups of SE’d workers prevailed in our research: first, some people were very unfamiliar with the social security system. This is consistent with several studies of developed economies, including Canada and Australia, and implies that eligible and entitled SE’d workers do not seek and claim compensation due to a lack of government-provided information about available programs [16, 61]. In this context, SE’d workers in this research suggested that the government run a rigorous social insurance literacy program using social and mass media. The second group in our study knew about these systems but decided not to opt into a formal support system. This is concerning as it suggests that the system was too complex to navigate. This ill-fit between policy and population needs need to be understood and addressed. In this context, our study reveals premium affordability, lack of SE-focused support programs, lack of trust in government systems, administrative challenges, discretion, confidence about savings, and relative affordability of private insurance. Most of the reasons mentioned above are not unique to Canadian SE’d workers. Countries with comprehensive social protection systems have similar limitations in protecting SE’d workers [62].

Although SE’d workers in this study underscored the bureaucratic challenges of claiming benefits from the government agencies, they did not focus on the issues related to employment misclassification or challenges with defining SE. However, a recent scoping review asserted that defining SE’d workers is a pressing challenge in most economically developed countries (e.g., Canada, Australia, USA, Denmark, UK) [12, 17, 63]. As well, their employment status can be vague in policy and legal documents [18]. Undoubtedly, the definition of SE’d is currently one of the constraints to protecting better SE’d workers against the backdrop of evolving work arrangements [14].

Interestingly, many SE’d workers showed positive attitudes towards opting into formal support systems after being informed about them by the interviewer. They were highly interested in having access to social security because they experienced insecurity and precarity with their work and income and had no easy access to government or privately regulated support systems. Several other studies have also found that job and income insecurity creates psychological distress and anxiety among workers [64]. SE’d workers in this study singled out the challenging bureaucratic aspects of benefit claims and related complications in terms of claiming procedures and fitting into eligibility criteria. As mentioned above, after participants were informed about the social security systems (e.g., income support benefits for sickness and family care, or EISB), they showed a positive attitude towards programs. However, no single participant we found in this study opted into this program, and very few had even a vague understanding of it. Given this context, there is a gap between policy and programs and the implementation of the policy or programs.

Our discussion, based on the findings, advances a central question: Why did SE’d people in this study not opt into the available government programs (e.g., income support benefits for sickness and family care)? If bureaucratic issues are there, governments may need to revisit their policy implementation strategies. In addition to gaps between policy and practice, this study reminds us that reforms of benefit coverage will not adequately protect SE’d workers unless the constraints, including premium affordability and knowledge gaps, are resolved. Given this backdrop, although people may have different views regarding mandatory or optional social security programs, in our view, the necessity of income support might outweigh the issue of ‘choice’ (mandatory or optional). In this case, the Canadian Government might consult the European Commission’s proposed mandatory social protection. This proposes that people, regardless of employment status, should come under the mandatory social protection coverage, but it will be means-tested [62].

In our study, although SE’d workers castigated the existing government-regulated programs due to their partial and limited coverage (workers’ compensation, health insurance), faulty eligibility assessment (workers’ compensation, welfare disability benefits), and costly premiums (income support benefits for sickness and family care), they appreciated Covid-19’s emergency response programs (e.g., COVID emergency support benefit) as successful and effective in addressing issues of SE’d workers. However, these programs are dogged by limitations such as moral hazard (i, e., some people have a tendency to abuse/misuse government funding, as it is free) and financial unsustainability (i.e., the COVID emergency support benefit might be excellent programs during the pandemic, but it is not a sustainable solution because people might have to stop working due to illness in a regular time). This is consistent with studies related to the effectiveness of social security programs during Covid-19 from other welfare states [65–68]. Despite these limitations, Canada’s goodwill in terms of successfully protecting SE’d workers during pandemic was comparable with OECD countries [68].

In this study, participants experienced that, due to limited health coverage by government health insurance, they had to spend out of their pocket for medications, diagnosis, eye examinations, therapies, and many more. In turn, they were sometimes forced to depend on loans or credit cards to stay afloat when ill or injured, which sometimes pushed them into the cycles of loans and poverty. Studies from other countries show that Canadians are not alone with health-related financial strain. Many economically developed countries, including the UK, Canada, United States, Australia, and New Zealand, have been cutting their state funding for health and health care supports every year [69]. All countries, across both less and more advanced economies, have been forming policies in line with neoliberal mindsets. That is, policies have been geared to populations as not being reliant on government supports and as needing freedom. Neoliberal ideas have shaped the mindset of people by encouraging self-dependence (e.g. personal savings) rather than dependent on state hand-outs. Of importance, this political game is played with young people, leaving them at risk of becoming trapped in unprotected forms of work (e.g., gig workers)[3]. In our study, SE’d workers shared concerns about compromising basic needs, such as shelter, food, education, recreation, health services, and medications, because of insufficient income and social protections. Hence, their right to a decent/quality life was adversely affected by their SE’d status. SE’d in this study questioned existing social security systems in terms of equity and social justice. They believed that they were not equally treated in terms of support systems, compared to salaried workers, despite the fact that they contribute to the economy as their employee counterparts do. In this context, their accounts represented a call for ‘restorative justice’ in terms of fair treatment of work and benefits. Accordingly, the ILO proposed Universal Labour Guarantee (ULG), which will be applied to all workers regardless of their contractual arrangements or employment status, and, therefore to SE’d workers as well. In our study, some SE’d workers called for universal guaranteed income (UGI) to protect them, in times of sickness, injury, or job loss. While supporters of UBI contend that everybody has the right to food and shelter based on redistributive justice, skeptics believe that UBI will decrease workers’ incentive to work [43]. European research proposed reconciling these conflicting views that social protections should be ‘decoupled’ from employment [3]. Instead, it should be linked to a ‘safety net’ for lower-income individuals [3]. Our study suggests that a privately arranged income security plan can leave many SE’d workers unprotected because many low-earning SE’d workers cannot afford the premiums. In this context, in agreement with many scholars, we emphasize the need for social insurance systems to cover all workers regardless of their employment status because underpinning private insurance and savings arrangements will likely widen the protection gaps, increase poverty, and exacerbate the inequality [3, 70, 71]. In addition to ILO’ assertions, Canada has an obligation to create an equal social protections system because it ratified ILO’s convention 111(“any distinction, exclusion, or preference … which has the effect of nullifying or impairing equality of opportunity or treatment in employment or occupation”, … “any distinction, exclusion or preference in respect of particular job based …”). In addition, support for SE’d people can also be found in the European Commission statement (2019): “The future of work demands the development of equitable, inclusive, and sustainable social protection systems, which ensure protection to meet people’s needs over the life cycle” [3] p.207. Similarly, Canada needs to address the protection of SE’d workers because it is committed to UN, which is implied in the three goals of UN Sustainable Development Goals (SDG): Goal 3: Good health and well-being for people, Goal 8: Decent work and economic growth, Goal 10: Reducing inequalities.

## Conclusion

To date, there has been little understanding of how to solo SE’d workers experience and navigate their health and work following illness, injury, or income reduction or loss. What are the existing formal support systems that SE’d workers can seek and use? Although scholars have previously engaged with existing statuary or formal support systems for SE’d workers, little is known about the experiences of solo SE’d workers regarding how they navigate their work, health, illness, or injury with the existing formal support systems. In this study, SE’d workers described encountering several constraints regarding access to formal support systems: premium affordability, information/knowledge gap, lack of SE social support programs, administrative challenges, confidence about savings, and lack of trust in government-regulated system. They also criticized the government-regulated formal support systems (publicly funded health care insurance plan, disability income support, and employment insurance for the SE’d) on the grounds of partial coverage, vague and intricated eligibility issues, and affordability. Although they appreciated receiving COVID-related government income support, some SE’d workers had reservations about the emergency programs because of weak management.

While we cannot recommend a cookie-cutter solution to better protect SE’d workers when they cannot work and earn an income, this study points to ways that although ‘Employment Insurance Special Benefits’ in Canada are not always used by SE’d workers, possibly due to the financial burden of premium payments, it nonetheless provides an example of a coverage system for SE’d workers that provide temporary income supports for parental, sickness, or compassionate support leave, etc. This is one way in which SE’d workers are recognized as a cohort of workers who are deserving of support during difficult economic periods. In all, it is encouraging to see in Canada that SE’d are recognized in these policies as it may create room for them to be covered by other national/provincial programs too, such as workers’ compensation and employment insurance. Secondly, in our study, many SE’d workers were unaware of the available support systems to which they were entitled, and this may be a widely prevalent situation among SE’d workers. Given this possibility, a social support literacy campaign may be introduced using mass media or social media. Then, we would also recommend that statutory social protection programs should be uncoupled from the employment benefits. Instead, governments might introduce a comprehensive program that may compensate or protects workers irrespective of employment status. For example, we could encourage social insurance systems instead of private insurance plans because private insurance usually requires higher premium payments than pooled social or group insurance. Furthermore, basic income policies may be a solution to providing a basic social safety net to SE’d people, among others. An advantage of this approach is that it draws on the general tax fund rather than relying on taxing incomes of low-wage SE’d people, who are already income insecure [43, 72]. In a sense, all are workers with their only asset of human capital; thus, all workers who depend on the sale of their capacity to work and survive should be covered and protected by labour protections and social supports (Fudge, 2003). This builds on my study participants’ suggestions that universal guaranteed income (UGI) would be a good mechanism to protect them when experiencing sickness, injury, or job loss. Finally, a recent Canadian study, based on 2016 census and tax data, revealed that gig workers among all workers in Canada rose from 5.5% in 2005 to 8.2%. According to the “2021 Canadian Self-employment Report”, SE is also heading to new trends in the post-Covid-19 labour market: of the 30 million working Canadians, nearly 7 million are expecting to make the jump to SE within the next two years, and the higher rate is even pronounced for those SE’d people who are under the age of 35 years [73]. Given this backdrop, we would recommend that governments take a special focus on young people who are SE’d. As this cohort is the future resource of Canadian labour market, their health and well-being are of paramount importance, and needs to be addressed in public policies, including social security, health policy, and labour market policy. Overall, SE’d workers as growing working populations require the consideration of equitable, inclusive, and sustainable social protection systems that ensure protection to meet people’s needs over the life cycle.

## Data Availability

The datasets used and/or analyzed during the current study are available from the corresponding author on reasonable request.

## List of Abbreviations

SE: Self-employment
SE’d: Self-employed
EI: Employment Insurance
EISB: Employment Insurance Special Benefits
CERB: Canadian Emergency Response Benefit
OHIP: Ontario Health Insurance Plan
ICT: Information and communication Technology
WSIB: Workplace Safety and Insurance Board
CRA: Canada Revenue Agency
ODSP: Ontario Disability Support Program
ULG: Universal Labour Guarantee
CESB: Canadian Emergency Student Benefit
IT: Information Technology
ILO: International Labour Organization
WHO: World Health Organization
UGI: Universal Guaranteed Income
UBI: Universal Basic Income
SDG: Sustainable Development Goals
UN: United Nations
OECD: Organization for Economic Cooperation and Development
CTP: Compulsory Third Party
SIRA: State Insurance Regulatory Authority
NDIS: National Disability Insurance Scheme
CEIS: Canada Employment Insurance Commission

## References

[1] Law Commission of Ontario : Vulnerable workers and precarious work: final report. 2012. [https://www.lco-cdo.org/en/our-current-projects/vulnerable-workers-and-precarious-work/vulnerable-workers-and-precarious-work-final-report-december-2012/]

[2] Wall S (2015). Dimensions of precariousness in an emerging sector of self-employment: a study of self‐employed nurses. Gend Work Organ.

[3] Behrendt C, Nguyen QA (2019). Ensuring universal social protection for the future of work. Transfer: Eur Rev Labour Res.

[4] Bögenhold D (2019). From Hybrid Entrepreneurs to Entrepreneurial Billionaires: observations on the socioeconomic heterogeneity of self-employment. Am Behav Sci.

[5] Weil D (2019). Understanding the Present and Future of Work in the fissured workplace context. RSF: The Russell Sage Foundation Journal of the Social Sciences.

[6] Quinlan M (2015). The effects of non-standard forms of employment on worker health and safety.

[7] Taylor M, Marsh G, Nicol D, Broadbent P. Good work: The Taylor review of modern working practices. 2017.

[8] ILO. : World employment social outlook. 2015. [https://www.ilo.org/global/research/global-reports/weso/2015/lang--en/index.htm]

[9] Labour Statistics at a glance, Self-employed Canadians: Who and Why? [https://www150.statcan.gc.ca/n1/pub/71-222-x/71-222-x2019002-eng.htm]

[10] Super and the self-employed. In.; 2016. [https://www.superannuation.asn.au/ArticleDocuments/359/Super-and-the-Self-Employed-May2016.pdf.aspx?Embed=Y]

[11] Clare R, Craston A. Super and the self-employed.In.; 2016. [https://www.superannuation.asn.au/ArticleDocuments/359/Super-and-the-Self-Employed-May2016.pdf.aspx?Embed=Y]

[12] Sharp L, Torp S, Van Hoof E, de Boer A (2017). Cancer and its impact on work among the self-employed: a need to bridge the knowledge gap. Eur J Cancer Care.

[13] Facey ME, Eakin JM (2010). Contingent work and ill-health: conceptualizing the links. Social Theory & Health.

[14] OECD Employment Outlook. 2019: The Future of Work [ 10.1787/9ee00155-en]

[15] The Effects of Self and Temporary Employment on Mental Health. : The Role of the Gig Economy in the UK. [20. https://ssrn.com/abstract=3395144 ]

[16] Quinlan M (2004). Workers’ compensation and the challenges posed by changing patterns of work: evidence from Australia. Policy and Practice in Health and Safety.

[17] Khan TH, MacEachen E, Dunstan D (2022). What Social supports are available to self-employed people when Ill or Injured? A comparative policy analysis of Canada and Australia. Int J Environ Res Public Health.

[18] Khan TH, MacEachen E, Hopwood P, Goyal J. Self-employment, work and health: A critical narrative review.Work2021(Preprint):1–13. 10.3233/WOR-213614 34744041

[19] ILO. : Ensuring better social protection for self-employed workers. In. Saudia Arabia: International Labour Organization (ILO) Organisation for Economic Co-operation and Development (OECD); 2020.

[20] Practices GWTTRoMW. : The Taylor Review of Modern Working Practices In.; 2017. [https://assets.publishing.service.gov.uk/government/uploads/system/uploads/attachment_data/file/679831/2018-02-06_Agencyworkerconsultationdoc_Final.pdf]

[21] Nordenmark M, Vinberg S, Strandh M (2012). Job control and demands, work-life balance and wellbeing among self-employed men and women in Europe. Vulnerable Groups & Inclusion.

[22] Kautonen T, Kibler E, Minniti M (2017). Late-career entrepreneurship, income and quality of life. J Bus Ventur.

[23] Rietveld CA, Van Kippersluis H, Thurik AR (2015). Self-employment and health: barriers or benefits?. Health Econ.

[24] Nordenmark M, Hagqvist E, Vinberg S. Sickness Presenteeism among the Self-employed and Employed in Northwestern EuropedThe Importance of Time Demands. *framework* 2019, 8:19. 10.1016/j.shaw.2019.01.003 PMC6598842 31297286

[25] Arnold NL, Ipsen C (2005). Self-employment policies: changes through the decade. J Disabil Policy Stud.

[26] Ashley D, Graf NM (2018). The process and experiences of self-employment among people with disabilities: a qualitative study. Rehabilitation Couns Bull.

[27] Fossen FM, König J (2017). Public health insurance, individual health, and entry into self-employment. Small Bus Econ.

[28] Hartman E, Oude Vrielink H, Huirne R, Metz J (2003). Sick leave analysis among self-employed dutch farmers. Occup Med.

[29] McNaughton D, Symons G, Light J, Parsons A (2006). My dream was to pay taxes”: the self-employment experiences of individuals who use augmentative and alternative communication. J Vocat Rehabil.

[30] Vermeylen G, Wilkens M, Biletta I, Fromm A. Exploring self-employment in the European Union. Publications Office of the European Union; 2017.

[31] Lewchuk W, Clarke M, De Wolff A (2008). Working without commitments: precarious employment and health. Work Employ Soc.

[32] Benavides FG, Silva-Peñaherrera M, Vives A (2022). Informal employment, precariousness, and decent work: from research to preventive action. Scand J Work Environ Health.

[33] Dahl MS, Nielsen J, Mojtabai R (2010). The effects of becoming an entrepreneur on the use of psychotropics among entrepreneurs and their spouses. Scand J Public Health.

[34] Stephan U, Roesler U (2010). Comparison of entrepreneurs’ and employee’s health in a national representative sample. J Occup Organizational Psychol.

[35] Bradley DE, Roberts JA (2004). Self-employment and job satisfaction: investigating the role of self‐efficacy, depression, and seniority. J Small Bus Manage.

[36] Tetrick LE, Slack KJ, Da Silva N, Sinclair RR (2000). A comparison of the stress–strain process for business owners and nonowners: differences in job demands, emotional exhaustion, satisfaction, and social support. J Occup Health Psychol.

[37] Vivekanandan B (2002). Welfare state system in Canada: emerging challenges. Int Stud.

[38] Khan TH, MacEachen E (2022). A furnished house without walls: examining the work and health support systems of self-employed workers in Ontario, Canada. Saf Health Work.

[39] Khan TH, MacEachen. Ellen, Dunstan, Debra: Self-employment and Social Supports in Canada and Australia: A Comparative Policy Analysis. In: *Canadian Association for Work & Labour Studies Conference: 2021; Alberta, Canada*: Canadian Association for Work & Labour Studies 2021.

[40] Khan TH. Self-employment, health, illness, and social security among solo self-employed workers. 2022. [https://uwspace.uwaterloo.ca/handle/10012/18939]

[41] Held P, Klassen BJ, Hall JM, Friese TR, Bertsch-Gout MM, Zalta AK, Pollack MH (2019). I knew it was wrong the moment I got the order”: a narrative thematic analysis of moral injury in combat veterans. Psychol Trauma: Theory Res Pract Policy.

[42] Ronkainen NJ, Watkins I, Ryba TV (2016). What can gender tell us about the pre-retirement experiences of elite distance runners in Finland?: a thematic narrative analysis. Psychol Sport Exerc.

[43] Ståhl C, MacEachen E. Universal Basic income as a policy response to COVID-19 and precarious employment: potential impacts on rehabilitation and return-to-work. In.: Springer; 2021. 10.1007/s10926-020-09923-w PMC7439237 32816204

[44] Martin D, Miller AP, Quesnel-Vallée A, Caron NR, Vissandjée B, Marchildon GP (2018). Canada’s universal health-care system: achieving its potential. The Lancet.

[45] Brassolotto J, Raphael D, Baldeo N (2014). Epistemological barriers to addressing the social determinants of health among public health professionals in Ontario, Canada: a qualitative inquiry. Crit Public Health.

[46] Bujacz A, Eib C, Toivanen S. Not all are equal: a latent profile analysis of well-being among the self-employed.Journal of Happiness Studies2019:1–20.

[47] Razavi S, Behrendt C, Bierbaum M, Orton I, Tessier L (2020). Reinvigorating the social contract and strengthening social cohesion: social protection responses to COVID-19. Int Social Secur Rev.

[48] Salonen J, Koskinen L, Nummi T (2020). The risk of under-insurance in the finnish statutory pension scheme for self‐employed workers: a trajectory analysis. Int Social Secur Rev.

[49] Conen W, Buschoff KS (2019). Precariousness among solo self-employed workers: a german–dutch comparison. J poverty social justice.

[50] Creswell JW, Poth CN. Qualitative inquiry and research design: choosing among five approaches. Sage publications; 2016.

[51] Khan TH, MacEachen E (2021). Foucauldian discourse analysis: moving beyond a social constructionist analytic. Int J Qualitative Methods.

[52] Khan TH, Raby R (2020). From missing to misdirected: young men’s experiences of sex education in Bangladesh. Sex Educ.

[53] Khan TH. Young men’s experiences and views of Sex Education in Bangladesh: a foucauldian discourse analysis. St. Catharines Brock University; 2018.

[54] Rodriguez MCG. “The stories we tell each other”: Using technology for resistance and resilience through online narrative communities. Emotions, technology, and health.edn.: Elsevier; 2016:pp. 125–147.

[55] Khan TH, MacEachen E. An Alternative Method of Interviewing: Critical Reflections on Videoconference Interviews for Qualitative Data Collection. *International Journal of Qualitative Methods* 2022, 21:16094069221090063.

[56] Patton MQ. Qualitative evaluation and research methods Thousnds Oaks. Canada Sage; 2001.

[57] Riessman CK. Narrative methods for the human sciences. Sage; 2008.

[58] Ståhl C, Karlsson EA, Sandqvist J, Hensing G, Brouwer S, Friberg E, MacEachen E (2021). Social insurance literacy: a scoping review on how to define and measure it. Disabil Rehabil.

[59] Llewellyn J, Howse RL. Restorative justice: A conceptual framework.Prepared for the Law Commission of Canada1999.

[60] Lynch JW, Everson SA, Kaplan GA, Salonen R, Salonen JT (1998). Does low socioeconomic status potentiate the effects of heightened cardiovascular responses to stress on the progression of carotid atherosclerosis?. Am J Public Health.

[61] Hilbrecht M (2016). Self-employment and experiences of support in a work–family context. J Small Bus Entrepreneurship.

[62] Jacqueson C (2021). Platform work, social protection and flexicurity in Denmark. Int Social Secur Rev.

[63] Grégoris M, Deschamps F, Salles J, Sanchez S (2017). Health assessment of self-employed in the food service industry. Int J Occup Environ Health.

[64] Watson B, Osberg L (2018). Job insecurity and mental health in Canada. Appl Econ.

[65] Fletcher M. Government’s income support responses to the Covid-19 pandemic.Policy Quarterly2020, 16(3).

[66] Schmid G (2020). Beyond european unemployment insurance. Less moral hazard, more moral assurance?. Transfer: Eur Rev Labour Res.

[67] Lord P (2020). Incentivising employment during the COVID-19 pandemic. The Theory and Practice of Legislation.

[68] Gosselin JS, Godbout L, Gagné-Dubé T, St-Cerny S (2020). Finances of the Nation: the economic response of governments in Canada to COVID-19 in the First Three months of the Crisis. Can Tax Journal/Revue fiscale canadienne.

[69] McGregor S (2001). Neoliberalism and health care. Int J Consumer Stud.

[70] Hossian MA, Khan TH. Poverty and public services: social exclusion of urban poor. Decelerated Decline, State of Poverty in Bangladesh. edn. Dhaka:Unnayen Onneshan, Shrabon Prokashani, Bangladesh;2012:pp. 113–134.

[71] Khan TH. Labour and Rights. *State of Labour in Bangladesh: Unnayan Onnesan* 2013.

[72] Pasma C, Regehr S. Basic Income: Some Policy Options for Canada. *rapport établi pour le Réseau canadien pour le revenu garanti* 2019.

[73] FreshBooks. Canadian Self-employment Report – 2021. In. United states 2021. [https://www.freshbooks.com/press/data-research/freshbooks-2021-canadian-self-employment-report]

